# Contextualizing learning to improve care using collaborative communities of practices

**DOI:** 10.1186/s12913-016-1566-4

**Published:** 2016-09-02

**Authors:** Lianne Jeffs, Julie McShane, Virginia Flintoft, Peggy White, Alyssa Indar, Maria Maione, A. J. Lopez, Sue Bookey-Bassett, Lauren Scavuzzo

**Affiliations:** 1 St. Michael’s Hospital, 30 Bond Street, Toronto, ON M5B 1 W8 Canada; 2 Keenan Research Centre of the Li Ka Shing Knowledge Institute, St. Michael’s Hospital, 30 Bond Street, Toronto, ON M5B 1 W8 Canada; 3 University of Toronto, 155 College Street, Toronto, ON M5T 1P8 Canada; 4 Canadian Health Outcomes for Better Information and Care (C-HOBIC), Ottawa, Canada; 5 Sunnybrook Health Sciences Centre, 2075 Bayview Avenue, Toronto, ON M4N 3 M5 Canada; 6 The Hospital for Sick Children, 555 University Avenue, Toronto, ON M5G 1X8 Canada

**Keywords:** Quality improvement, Interorganizational learning, Collaboration, Communities of practice, Mentorship, Point-of-care

## Abstract

**Background:**

The use of interorganizational, collaborative approaches to build capacity in quality improvement (QI) in health care is showing promise as a useful model for scaling up and accelerating the implementation of interventions that bridge the “know-do” gap to improve clinical care and provider outcomes. Fundamental to a collaborative approach is interorganizational learning whereby organizations acquire, share, and combine knowledge with other organizations and have the opportunity to learn from their respective successes and challenges in improvement areas. This learning approach aims to create the conditions for collaborative, reflective, and innovative experiential systems that enable collective discussions regarding daily practice issues and finding solutions for improvement.

**Methods:**

The concepts associated with interorganizational learning and deliberate learning activities within a collaborative ‘Communities-of-practice’(CoP) approach formed the foundation of the of an interactive QI knowledge translation initiative entitled PERFORM KT. Nine teams participated including seven teams from two acute care hospitals, one from a long term care center, and one from a mental health sciences center. Six monthly CoP learning sessions were held and teams, with the support of an assigned mentor, implemented a QI project and monitored their results which were presented at an end of project symposium. 47 individuals participated in either a focus group or a personal interview. Interviews were transcribed and analyzed using an iterative content analysis.

**Results:**

Four key themes emerged from the narrative dataset around experiences and perceptions associated with the PERFORM KT initiative: 1) being successful and taking it to other levels by being systematic, structured, and mentored; 2) taking it outside the comfort zone by being exposed to new concepts and learning together; 3) hearing feedback, exchanging stories, and getting new ideas; and 4) having a pragmatic and accommodating approach to apply new learnings in local contexts.

**Conclusions:**

Study findings offer insights into collaborative, inter-organizational CoP learning approaches to build QI capabilities amongst clinicians, staff, and managers. In particular, our study delineates the need to contextualize QI learning by using deliberate learning activities to balance systematic and structured approaches alongside pragmatic and accommodating approaches with expert mentors.

## Background

The use of interorganizational, collaborative approaches to build capacity in quality improvement (QI) in health care is growing and showing promise as a useful model for scaling up and accelerating interventions that bridge the “know-do” gap to improve aspects of clinical care and provider outcomes [1–9]. Fundamental to a collaborative approach is interorganizational learning whereby organizations acquire, share and combine knowledge with other organizations and have the opportunity to learn from the successes and failures of their peers in improvement areas [6]. This learning approach aims to create the conditions for collaborative, reflective, and innovative experiential systems that enable collective discussions regarding daily practice issues and finding solutions for improvement by integrating tacit-explicit knowledge [9, 10]. Interactive dialogue occurs by including participants in deliberate learning activities around The Model for Improvement, Plan Do Study Act (PDSA) small scale cycles and QI tools [11] and the application of learning to a QI project, in partnership with a mentor [4–6]. Using deliberate learning activities may enable working teams to absorb new practices and foster a deeper understanding of the practices required to ensure quality care [11].

The literature relative to the value of using interorganizational, QI collaboratives (QICs) is growing. However, there remains limited understanding as to what factors within the QIC approach are responsible for producing the desired outcomes [4]. Limited empirical knowledge exists on best practices for engaging point-of-care clinicians and non-physician staff in collaborative QI efforts [5, 12].

Further insight into the effectiveness of interorganizational collaborative approaches for QI may emerge from understanding the experiences of those who participate in these approaches. In this context, a qualitative exploration was conducted to elicit the perceptions and experiences of project leads, unit managers, mentors, point-of-care clinicians and staff who participated in the Knowledge Translation of Performance Data for Frontline Nurses and Leaders Initiative (PERFORM KT), an interorganizational, collaborative approach to building capacity for QI.

## Methods

### Intervention description

The concepts associated with interorganizational learning [4–6] and deliberate learning activities [11] within a collaborative ‘community of practice’(CoP) model formed the foundation of the development, implementation and evaluation of PERFORM KT [10, 13, 14]. Deliberate learning activities generate operational knowledge about how to perform new practices effectively as well as conceptual knowledge about cause-and-effect relationships that make practices effective [15]. Operational and conceptual knowledge together can increase a teams’ absorptive capacity for learning new practices, the ability to change and adapt routines to achieve their goals [11].

**Fig. 1:**
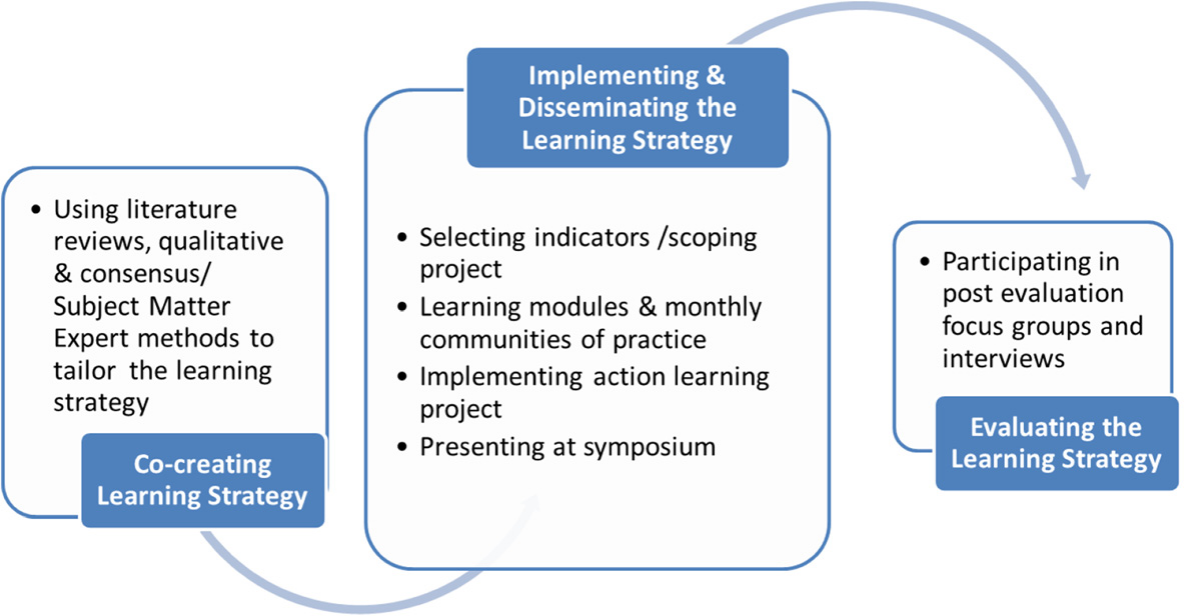
Learning Framework

In this context, PERFORM KT aimed to equip point-of-care clinicians, staff and clinical leaders with the knowledge and skills required to use clinical data to inform their day-to-day practice. Informed by the key concepts of interorganizational learning, QICs, communities of practice as well as interviews with nurse leaders, [16] PERFORM KT applied the knowledge gained from interactive learning and coaching and mentorship to QI projects implemented within each participating teams’ local clinical units. Five learning modules including making data relevant, interpreting data, using data to inform practice, translating data, and sustaining data, were co-created with expert mentors and representatives from the unit-level manager and point-of-care nurse cohorts. See Fig. 1.

### Teams

All health-care organizations (*n* = 10) which had participated in the preceding, related initiative, the National Nurse Quality Report, [17] were invited to participate in PERFORM KT. Three of the 10 organizations accepted the invitation. Two sent one team each and the third sent three. A fourth organization was invited and sent 4 teams for a total of 9 teams participating in PERFORM KT, 7 teams from two acute care hospitals, 1 from a long term care center located in another province, and 1 from a mental health sciences center.

Team leaders and members from each of the PERFORM KT projects (see Table 1) attended the monthly CoP learning sessions (*n* = 6). Between learning sessions the teams, with the support of an assigned mentor, implemented a QI project and monitored their results. The monthly CoP provided the teams and their mentors an opportunity to share their progress and challenges related to their QI projects in a safe, supportive environment. Partial financial support was available for the release time for nursing/clinical staff to be able to attend the monthly CoPs, work on their QI projects, and participate in an end of cycle symposium. As part of the translating knowledge learning module, teams were given tips on how to prepare and present a poster and slide presentation and engaged in a mock session of their oral presentation in preparation for the end of cycle symposium. Figure 1 provides the overall approach of PERFORM KT.

**Table 1:** Project titles

Pain it Forward on Respirology
Painless Documentation
Preventing Falls With Intentional Rounding General Surgery/GI/Plastics
Reducing Injuries Related to Falls
Aggressive Behaviour Minimization in Neuropsychiatry
First Line of Defence: Decreasing Medical Adhesive Related Skin Tears in the ICU
Enabling Occupational Engagement Through Management of Agitated Behaviors
Improving level of function with early mobilization for patients with fractures
Falls Prevention Through Improved Inter-professional Communication And Mixed Methodology

### Data collection

Interviewees were recruited from the nine participating teams using the following eligibility criteria: 1) employed at one of the participating units/sites; 2) involved in PERFORM- KT; and 3) able to provide informed consent. An initial e-mail was sent to the point-of-care clinicians and staff, mentors and managers who participated in the initiative to inform them of the evaluation study. PERFORM KT project leaders at the clinical unit level were introduced to the study by a research assistant during one monthly CoP session. Upon completion of their projects, PERFORM KT project leaders were invited to participate in a focus group interview. Data was also collected using one-on-one interviews with point-of-care clinicians and staff, mentors and managers. Research staff used a semi-structured interview guide consisting of a series of open-ended questions to direct and focus the discussion.

### Data analysis

Focus groups and individual interviews were audio-taped and transcribed verbatim. Data were analyzed using a directed content analysis approach [18]. Specifically, the principal investigator and two members of the research team independently reviewed each transcript line by line to identify codes from within the text and then met to develop the initial coding schema by consensus. Categories were then constructed to incorporate the codes and the coding schema was refined and reviewed by the principal investigator. An iterative analytical process was used, wherein the initial coding schema was applied to each transcript to further develop and refine themes, subthemes, and subcategories in efforts to capture variant occurrences within the data. Data collection ended when saturation of themes occurred. Throughout the data analysis phase, ongoing discussion among team members ensured that issues were systematically addressed.

## Results

### Sample characteristics

Four focus groups were conducted with 17 PERFORM project team participants, with an interview completed with one project team participant who could not attend the focus group. Focus groups 1, 2 and 3 consisted of nurses, while focus group 4 consisted of health disciplines staff. This was to accommodate participant preference to do the focus group at the end of their shift. A further 29 personal interviews were conducted with point-of-care nurses, unit managers and PERFORM team mentors. Demographic data was received from 42 of the 47 participants only. Of the focus group participants, 87 % had been in their role and 60 % had been with their organization for less than 5 years. The completed demographics forms indicate that the focus group participants were comprised of nurses (*n* = 11), occupational therapists (*n* = 2), physiotherapists (*n* = 1), and a unit clerk (*n* = 1). In addition, individual interviews were conducted with point-of-care nurses on units that were involved in PERFORM KT projects of whom 31 % had been in their current roles and in their organization for less than 5 years, while 36 % had been with their organization and in their roles for more than 20 years. Managers and mentors were more likely to have Masters preparation (80 and 100 %, respectively).

### Emergent themes

The following four key themes emerged from the narrative dataset around experiences and perceptions associated with the PERFORM KT learning strategy: 1) being successful and taking it to other levels by being systematic, structured, and mentored; 2) taking it outside the comfort zone by being exposed to new concepts and learning together; 3) hearing feedback, exchanging stories, and getting new ideas; and 4) having a pragmatic and accommodating approach to apply new learnings in local contexts.

#### Being successful and taking it to other levels by being systematic, structured, and mentored

This first theme reflects how study participants described the value of the systematic and structured approach of the PERFORM KT initiative. The participating project leads identified the benefit of the step-by-step approach to understanding QI methods and tools, and knowledge translation strategies. Interestingly, some participants attributed the success of their project and the ability to take their work to other levels such as, presenting to internal stakeholders and presenting nationally, to the PERFORM KT learning approach. Other participants articulated the benefit of having established timelines built in throughout PERFORM KT that participants were accountable to meet. As the PERFORM KT participants benefited from the structure of the formal education program, in turn they used a systematic and structured approach to guide their respective unit specific projects. The point-of-care clinicians and staff expressed appreciation for the various tools, education and process improvements implemented by their PERFORM KT participant colleagues which helped keep the units’ project focused and on target. For example, through their PERFORM KT class work, team leads created unit specific, project related tools to share with their unit colleagues. These tools included cue cards, a visual representation of 4 moments of assessment (modelled after the 4 moments of hand hygiene), a rounding schedule and skin tear bundle kits. This theme is further illustrated by the following quotes:

*We had a general idea of what we wanted to achieve, but the way it was introduced was that we needed more structure which was provided by PERFORM KT. How to keep everything regimented instead of us just doing whatever we wanted to- making it more of a systematic approach to even just see what the knowledge gaps were. If we had just gone ahead and done it the way we wanted to do it may not have been as successful as what it was. It may not have led to where we’ve taken it now to other areas so presenting it nationally, meeting with directors to take it hospital wide.* (Project Leader Focus Group 3)

*The cue cards they gave us [were] actually quite helpful when you ask the patient. At least they give you guidance.* (Point-of-care Nurse 19)

*The little tool they made a piece of paper with the four moments of assessment I think was really beneficial.* (Point-of-care Nurse 17)

Another key component of the PERFORM KT initiative that was perceived favourably by the project leaders and managers was the ongoing mentorship opportunities. The mentors were described by participants as keeping ‘things on track’ and ‘grounded’ from their experiences with various QI projects. Mentors kept their teams on track by being available, responsive, and knowing when to step in if they were struggling, and by helping teams scope their projects to achieve realistic and meaningful outcomes. As one project leader described the comfort in “*knowing that they were very close by and going to keep you on track”*. The next series of excerpts further highlight this theme.

*I would say having their ongoing mentorship was crucial to us having something concrete and meaningful to present today. Even helping us create goals for sustainability moving forward helps us keep grounded so we are not again reaching for those goals that are not really that achievable.* (Project Leader Focus Group 4)

*The mentors were equally responsive and then sent them information so they feel comfortable and they weren’t struggling and they could break it down for them to really understand.* (Manager 1)

*I think the mentors really kept them on track in terms of letting them know what was expected before coming to each of the communities of practice. Sort of being able to assign tasks and know that by the next week or the next CoP, this is what you need to have done.* (Manager 3)

#### Taking it outside the comfort zone by being exposed to new concepts and learning together

This second theme captures the tensions experienced by participants with the learnings associated with the PERFORM KT initiative. Although, the majority of participants described enjoying the learning sessions, most of the QI concepts (e.g. cause and effect fishbone, PDSA cycles, etc.) were new to them. For some participants (project leaders and managers) this caused anxiety and fear as they did not want to fail with their projects. In addition to QI concepts, project leaders were taught knowledge translation strategies which focused on how to disseminate project findings and insights for their peers and at the end of cycle symposium. Further, the project leads were learning these new concepts and had to then apply these learnings in rapid cycle changes with their colleagues within their respective clinical units.

*Make sure you always have two[communities of practice]. So you have the larger community of practice but to have the smaller CoP which was the two of you. I know nurse X has been involved in other QI initiatives, but nurse Y hadn’t had the background with the PDSA cycle.* (Project Leader Focus Group 2)

*What I picked up on was often we see a problem and want to fix it right away without doing the analysis piece, so I think I learned to more fully appreciate that piece.* (Project Leader Focus Group 1)

*Outcomes on the unit have been awareness of our issue as an issue, awareness of the QI process and I think, awareness too of organizational and unit goals, so examples of those are awareness of QI, getting people familiar with the idea of surveys and feedback and changing education, and trying again PDSA cycles, so awareness of organizational goals.* (Project Leader Focus Group 3)

In addition to the learnings of the project leaders, the managers and mentors also provided reflections on what they learned from the program. For example, some managers were anxious about how their staff was going to manage their projects and they wanted to ensure it was a positive experience for their units and participated in the monthly CoPs. Further, both new and experienced mentors identified the ability to work with a new group of people as a source of learning. For example the experienced mentor was quite fascinated with their initial view that *“everyone knows QI by now you think the message is out and…this is not news anymore and boy it sure is”.* More examples to support this theme are noted below:

*I found the content of the communities of practice really helpful, the introduction of basic concepts, of fish diagrams, and how to structure, how to breakdown work flow, stuff we are not exposed to.* (Project Leader Focus Group 3)

*Seeing that we’ve presented twice on the topic, it can give you some confidence to take it outside of our comfort zone. We didn’t meet our aim completely, that’s okay too.”* (Project Leader Focus Group 4)

*The participants were learning how to do performance improvement and work through their issues, I was learning how to be a mentor. We were all learning how to be mentors.* (Mentor 2)

*It was a bit scary at the beginning because you don’t want to fail. But they ensured that nobody was going to fail at this and there was always room to continue to grow.* (Manager 1).

#### Hearing feedback, exchanging stories, and getting new ideas

This third theme reflects how participants valued the CoP component of the PERFORM KT learning strategy. Project leaders, mentors and managers described the CoPs as a place to hear feedback, exchange stories of what was working well or not, and ‘get new ideas’ and ‘take on new angles’ on their respective QI projects between participating units and across the hospitals. Specifically, PERFORM KT participants spoke of generating new ideas from other project teams’ suggestions or successes and appreciated new perspectives offered by their peers. For the most part this was informal at the monthly CoPs, however at one CoP, the teams were paired up with another team outside their organization doing a similar project. One team presented their project and the paired team was responsible for giving feedback.

The other formal exchange was at the end of the cycle symposium which participants viewed as a beneficial opportunity to present updates, outcomes and insights associated with participation in the PERFORM KT initiative and their respective local QI projects. One project leader described *“the ability to see the creativity out there and different solutions”* as inspiring. Further, PERFORM KT participants also appreciated that they were not alone in the successes and challenges of their QI journey. The following series of narrative excerpts that illustrate this theme are noted below:

*I took notes and “I didn’t think of that”, so I got some new ideas or some new takes on different angles of what we could do back home on our particular project.* (Project Leader Focus Group 1)

*I think also what was good was the CoP where the teams got to talk with each other and they were able to see where other teams were at, and what challenges they were having and they were able to share their successes and challenges.* (Mentor 4)

*It was really really nice for our groups to see what the others had done. I don’t know how anybody else felt, but I thought it was a proud moment because we were talking about different things and you got to see what everyone was doing. It was really nice. I loved the Symposium.* (Manager 2)

*I really enjoyed hearing people’s feedback and what they’ve been going through and some of the challenges that they’ve run into. It’s helpful to know you’re not the only person running though those challenges.* (Manager 3)

#### Having a pragmatic and accommodating approach to apply new learnings in local contexts

This fourth theme captures the experiences described by study participants (mainly project leaders and the point-of-care nurses) associated with project leaders pragmatic and flexible approaches to engage their unit colleagues in their respective QI projects. Point-of-care clinicians and staff identified that the pragmatic and practical approach that was employed by project leaders with a focus on a clinically relevant topic resonated with them. Further, the participants interviewed also valued that the accommodating nature of the project leaders when they were engaging their colleagues in QI efforts, particularly amidst managing clinical priorities in daily practice. Engaging point-of-care nurses was often done informally and at times convenient for point-of-care nurses. The following quotes illustrate this theme:

*You could see the practical value of it and nurses are very pragmatic.* (Point-of-care Nurse 13)

*They made adjustments based on people’s feedback and followed up. When, and when there was questions about it, there was someone there to answer.”* (Point-of-care nurse 5)

*They found a time to do the sessions when we are actually not busy. Instead of us accommodating them, they accommodated us which is number one with nursing because when we’re doing nursing care, you can give us a 15 minute session but if they’re calling in the middle of a busy session, it’s not going to get in here.”* (Point-of-care Nurse 18)

*Our falls prevention, what’s really critical is we have this database now on our falls so we see the changes right away. We know exactly, is what we are doing is it working? We have a review with our staff meeting every week and we review a fall and it involves all the staff giving feedback. It’s automatic involvement and engagement and understanding whether what we’re doing is actually working.* (Project Leader Focus Group 2)

*If you have ideas, you can enlighten other staff and bring change, so we had a lot of staff curious about the project itself, how you got your funding for it, because they had so many ideas themselves of different QI or research projects that they wanted to find a way to get developed on their units as well.* (Project Leader Focus Group 3)

## Discussion

Our study findings provide insight into what participating point-of-care clinicians, staff, unit managers, and mentors perceived to be important active ingredients of the inter-organizational collaborative learning approach to guide their local QI efforts. Collectively our findings add to a small yet important emerging body of evidence that aims to understand the specific features within organizational and system contexts and active ingredients of QI efforts that drive change with point-of-care clinicians and staff to enhance effective and efficient care.

Our study highlights the importance of having a systematic, structured interorganizational collaborative learning approach in concert with being pragmatic and accommodating when applying learnings at the local clinical unit level. This exemplifies the goals of deliberate learning for building QI capabilities for point-of-care clinicians and staff [11, 15]. Of particular relevance for participants were the structured learning modules and resource binders that enabled them to understand and apply key QI, knowledge translation and change management concepts and tools into practice in a timely, adaptable fashion. The key role of a structured approach to QI education has also been previously reported [4, 5, 19]. Part of the structured learning modules included exposing the project leads at the clinical level and the managers to the larger QI and patient safety field or as one participant described “seeing the bigger picture”. This is consistent with a study that found that participation in QI training expanded participants’ QI knowledge and skills and enabled them to put the “pieces of the puzzle” together [19].

Our finding around the key role of the mentors in working with their assigned teams to provide expertise, keep things on track, and overcome barriers is consistent with other QI education interventions [19–21]. Our study further delineates the role of the mentor as they worked with their assigned teams to initially explore the cause and effect of their respective topic areas using QI tools (e.g. Ishikawa fishbone cause and effect, process mapping) and then apply learnings to their respective local QI projects. In this capacity they served as “sense-makers” as they were able to impart to the teams an understanding of the operational and conceptual knowledge required to perform new practices (applying QI concepts locally and participating in knowledge translation strategies at the end of cycle symposium) effectively. In turn, the teams were able to challenge some of their original assumptions and adapt their focus to achieve their goals and learn to be outside their comfort zone.

Our finding around how participants described the interactive dialogue that took place during the monthly meetings as inspiring, motivating and creative adds support to the value of learning through interorganizational learning [5, 6, 10, 11] and collaborative CoP [13, 14] approaches. By sharing both successes and failures, teams were able to learn valuable dos and don’ts and consider practices that might be worthy of adopting as part of their QI efforts [6]. In our study, participants identified that value of having opportunities to provide and receive both formal, structured and informal feedback. Finally, participants also identified the objective nature of feedback provided by others from outside their institution for example, not feeling they are the only ones dealing with QI issues and the challenges associated with implementing QI projects into local clinical contexts.

Study findings can be used to guide future collaborative, inter-organizational CoP learning approaches to build QI capabilities amongst point-of-care clinicians, staff, and managers. Future educational strategies should consider the balancing of learning to be systematic and structured alongside pragmatic and accommodating depending on the context. Key to this balanced approach is being responsive to learning styles and different perspectives within a safe space for learning [22]. Further, inclusion of expert mentors who serve as “sense makers” and work with point-of-care clinicians, staff, and managers as they move in and out of their comfort zones is paramount. Study findings may also offer insights into the “active ingredients” of collaborative, inter-organizational communities of practice learning approaches to build QI capabilities amongst point-of-care clinicians, staff, and managers.

Further exploration on how best to contextualize learning to improve care within collaborative CoP approaches and examination of outcomes associated with this approach on a larger scale in Canada and beyond is recommended. Future research could build on this empirical work and mixed methods approaches used to evaluate similar QI collaborative efforts - Transforming Care at the Bedside [9] and the Betty Irene Moore Nursing Initiative [23]. Specifically, multi-national, multi-site research that employs more rigorous experimental designs that include system, professional, organizational, team-based and patient oriented outcomes and experiences are required.

### Study limitations

The following limitations need to be taken into consideration when interpreting our study. The first limitation is the potential for social desirability bias from the participants and the self-report nature of the interviews and focus groups. In particular the participating project leaders from the clinical units and unit managers may have been supportive of learning and professional development opportunities. The second limitation is around transferability of findings given that the data was only collected from a small sample size drawing from four healthcare organizations with three in Ontario and one in Manitoba.

## Conclusion

Our study findings contribute to the evolving literature around the value of using QI collaboratives;[1–4, 7–9] interorganizational learning [5, 6, 10, 11] and collaborative communities-of-practice [13, 14] to build QI capabilities amongst point-of-care clinicians, staff, and managers. Study findings offer insights into the active ingredients of collaborative, inter-organizational communities of practice learning approaches to build QI capabilities amongst point-of-care clinicians, staff, and managers. Of particular note, our study delineates the need to contextualize QI learning by using deliberate learning activities to balance systematic and structured approaches alongside pragmatic and accommodating approaches with expert mentors. Future studies should be conducted to further explore how best to contextualize learning to improve care within collaborative CoP approaches.

## Abbreviations

QI, quality improvement; CoP, communities of practice; PERFORM KT, knowledge translation of performance data for frontline nurses and leaders initiative
